# Variable delay-to-signal: a fast paradigm for assessment of aspects of impulsivity in rats

**DOI:** 10.3389/fnbeh.2013.00154

**Published:** 2013-10-23

**Authors:** Hugo Leite-Almeida, António Melo, José M. Pêgo, Sara Bernardo, Nuno Milhazes, Fernanda Borges, Nuno Sousa, Armando Almeida, João J. Cerqueira

**Affiliations:** ^1^Life and Health Sciences Research Institute (ICVS), School of Health Sciences, University of MinhoBraga, Portugal; ^2^ICVS/3B's - PT Government Associate LaboratoryBraga/Guimarães, Portugal; ^3^CIQUP/Departamento de Química e Bioquímica, Faculdade de Ciências, Universidade do PortoPorto, Portugal; ^4^Instituto Superior de Ciências da Saúde-NorteGandra, Portugal

**Keywords:** rodent behavior, decision impulsivity, response impulsivity, delay-discounting, 5-csrtt, methamphetamine, MK-801

## Abstract

Testing impulsive behavior in rodents is challenging and labor-intensive. We developed a new behavioral paradigm—the Variable Delay-to-Signal (VDS) test—that provides rapid and simultaneous assessment of response and decision impulsivity in rodents. Presentation of a light at variable delays signals the permission for action (nose poke) contingent with a reward. 2 blocks of 25 trials at 3 s delay flank a block of 70 trials in which light is presented with randomly selected 6 or 12 s delays. Exposure to such large delays boosts the rate of premature responses when the delay drops to 3 s in the final block, an effect that is blunted by an acute methamphetamine challenge and that correlates with the delay-discounting (DD) paradigm (choice impulsivity). Finally, as expected, treatment with the NMDA antagonist MK-801 caused a generalized response increase in all VDS blocks. The pharmacological validation, particularly with methamphetamine which has a well established dual effect on response and decision impulsivity, and the correlations between the impulsive behavior in the DD and VDS paradigms, suggests that the later is able to provide, in a single session, a multi-dimensional assessment of impulsive behavior.

## Introduction

Impulsivity is defined as a tendency to act prematurely without foresight (Dalley et al., 2011). It is a non-unitary construct embracing impulsive response and impulsive choice (Evenden, 1999b; Winstanley et al., 2006; Dalley et al., 2011; Dalley and Roiser, 2012). This multifactorial trait depends on a complex morphophysiology involving multiple brain areas and neurotransmitter systems (Dalley et al., 2011; Dalley and Roiser, 2012). Impulsivity is part of the normal behavioral repertoire, but, when out of the normal range, can result in a disruptive behavior, encountered in several psychiatric disorders including obsessive-compulsive disorder (OCD), attention deficit/hyperactivity disorder (ADHD), mania, substance abuse, and schizophrenia (Evenden, 1999a,b; Moeller et al., 2001).

Impulsive behavior is also present in rodents, both in normal conditions and in models of psychiatric disease (Adriani et al., 2003; Huskinson et al., 2012; Pattij and De Vries, 2013). It has been assessed in a number of paradigms that are well established in terms of their face, construct and predictive validity, with the go/no-go (Harrison et al., 1999), the stop-signal reaction task (SSRT; Eagle et al., 2008), the 5-choice serial reaction time task (5-csrtt; Carli et al., 1983) and the delay-discounting (DD; Evenden and Ryan, 1996) among the most used (for review see Winstanley et al., 2006; Dalley and Roiser, 2012). The construct of each of these paradigms varies substantially, reflecting the non-unitary characteristic of impulsivity (Winstanley et al., 2010; Dalley et al., 2011; Dalley and Roiser, 2012). In the first three paradigms, impulsive responses result from an inability to refrain from an action either when waiting for a go signal or in the presence of an explicit no-go signal, reflecting what is considered “response impulsivity.” In contrast, in the DD, impulsive responses are the result of a deliberate choice between a maximal, though delayed, and an immediate but small, reward, reflecting a so-called “decision impulsivity”. Although these paradigms have provided valuable tools to study impulsivity in rodents, they present several limitations including the extensive time commitments (spanning over 2 months in some cases), the possibility of confounding by factors like attention and reward valuation, the acquisition of repetitive behaviors (accommodation) due to the sequential performance of the paradigms and the mono-dimensionality of the construct assessed in each test, that limits the behavioral readouts to a single type of impulsivity.

In order to circumvent some of these problems, we developed a new behavioral paradigm, the Variable Delay-to-Signal (VDS) test, consisting of a series of trials, in a single 30 min session, in which the time period (60 s) where an action (nose poke) triggers the delivery of a sugared reward is signaled by a light, presented after a variable delay; a block of 3 s delay trials is followed by a block with large and variable delays (randomized between 6 and 12 s) before a final block again with a 3 s delay. Rats learn the operant sequence (nose poke/reward) rapidly (after a few trials) and the entire protocol lasts for 10 days, a significant reduction comparing to previously described paradigms. In addition, we have validated the VDS by employing two drugs with well-established actions on impulsive behavior, methamphetamine and MK-801, and by comparing the individual performance against two reference paradigms, 5-csrtt (response impulsivity) and DD (decision impulsivity). Given the observed dual pro- and anti-impulsivity action of methamphetamine (Hayton et al., 2012) in different components of the VDS and their correlations with the reference paradigms, we suggest that the VDS provides an effective assessment of both response and decision impulsivity.

## Materials and methods

### Organization of the experimental procedures

Three months old, male Wistar Han rats (Charles River Laboratories, Barcelona, Spain) were used in all experiments. Animals were kept in a room with controlled temperature (22 ± 1°C), 12 h light/dark cycle (lights on at 8 a.m.) and housed in pairs in plastic cages with food and water available *ad libitum*. The dietary regimen was restricted to 1 h of food availability (19:00–20:00) 3 days before the initiation of the behavioral experiments. Body weight was thereon controlled to ensure that it did not go below 85% of the initial value. All procedures involving animals were approved by local authorities and the experiments were performed according to the guidelines of European Community Council Directive 2010/63/EU.

**Figure 1:**
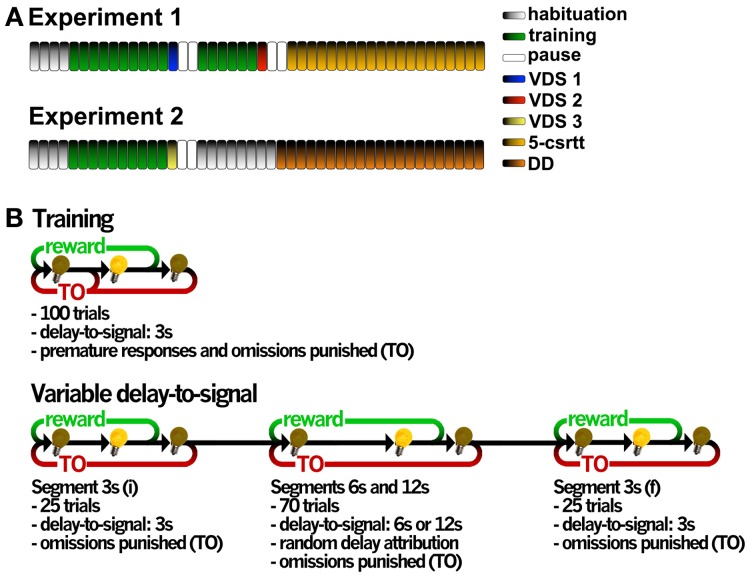
**General organization of the validation experiments and operational diagrams of the VDS and preceding training protocol. (A)** In experiment 1, two VDS protocols were performed in the same group of animals to test for methamphetamine and MK-801 effects on impulsivity, followed by the 5-csrtt performed in drug-free conditions during the whole protocol. In experiment 2, VDS was followed by DD. Each bar represents a session, except for 5-csrtt and DD whose protocol extension varied between individuals. **(B)** The VDS consisted in two parts: the training protocol (10 sessions) and the VDS proper (1 session); while in the first the delay-to-signal was fixed (3 s) and pre-signal nose pokes were punished (TO), in the VDS proper, 2 blocks of 25 trials at 3 s delay were interposed by a block of 70 trials at 6 and 12 s, pre-signal responses were registered but not punished with a TO. In both training and VDS the signal duration was set to a maximum of 60 s; the absence of a response within this period was registered as an omission. 5-csrtt—5 choice serial reaction time task; DD—delay discounting; TO—timeout; VDS—variable delay-to-signal task.

We conducted two independent experiments (Figure 1A): in the first, 20 animals sequentially performed the VDS under methamphetamine/vehicle (VDS 1), the VDS under MK-801/vehicle (VDS2) and the 5-csrtt; in the second, 12 animals without any treatment performed the VDS (VDS3) followed by the DD. In both the VDS1 and VDS2, animals were assigned to receive either drug or vehicle according to their performance in the preceding training sessions, so that both groups had a similar mean prematurity score; in addition, in VDS2 both vehicle and MK-801 groups had a similar number of animals previously treated with methamphetamine. In VDS 1 session two animals (one from each group) have not finished the task probably due to a failure in the reward delivery system; these were excluded from further analyses.

### Drugs

Methamphetamine was used in VDS 1 session to lessen impulsive behavior. A dose of 1 mg/Kg of the racemic mixture (effective dose of 0.5 mg/Kg) was administered intraperitoneally in a freshly prepared solution at a concentration of 1 mg/mL (in saline) 30 min before the initiation of the session (Hayton et al., 2012). Methamphetamine hydrochloride was synthesized by an adaptation of a previously described method (Milhazes et al., 2007). The NMDA antagonist MK-801 was used in VDS 2 session to boost impulsive behavior. A dose of 0.03 mg/Kg was administered intraperitoneally in a freshly prepared solution at a concentration of 0.03 mg/mL (in saline) 10 min before the initiation of the session (Fletcher et al., 2011). MK-801 was obtained from Calbiochem (CA, USA, Catalog Number: 475878-10MG).

### Variable delay-to-signal

The VDS task was performed in square operant chambers (OC; 25 × 25cm; TSE Systems, Germany) having on a curved wall 5 squared apertures (2.5 cm) elevated 2 cm from the grid floor and, in the opposite wall, a similar aperture (food magazine) connected to a pellet dispenser. Each aperture contained a 3W lamp bulb and an infrared beam system to detect the activity of the animal. Three 5-hole OCs, placed within sound attenuating boxes with individual electrical fans for ventilation and white noise production, were simultaneously used in our studies.

Animals were habituated to the testing conditions in 4, twice daily (am/pm) sessions, 5h apart. In the first 2 sessions, animals were placed in the OC for 15 min with all lights off, access to apertures 1–5 blocked by metallic caps and 10–15 sugared pellets (45 mg, Bioserv Inc., New Jersey, EUA) available in the food magazine. In sessions 3–4, animals were placed in the OCs for 30 min, all lights were on and animals had free pellets available in the center nose poke aperture (response aperture, 2–3) and the food-magazine (10–15). The protocol for VDS was initiated the following day and included two phases, i. training and ii. test (Figure 1B).

- *Training* Sessions were initiated by turning on the house light and delivering one sugared pellet in the food magazine, the collection of which started an intertrial interval (ITI) of 3 s to allow the animal to ingest it. Trials then started, consisting of a 3 s period with only the house light on (delay period), followed by lightning of the response aperture for 60 s (response period). Nose pokes in this aperture were either punished with a timeout (TO) period in complete darkness (5 S), if performed during the delay period (premature responses), or rewarded with the delivery of a pellet if performed during the response period. Collection of a food reward always triggered a 3 s ITI, before a new trial begun. Except for the TO periods, the house light was always on. Each session comprised 100 trials to be performed in a maximum of 30 min. Training sessions occurred twice daily, with a 5 h interval in between, for 5 consecutive days. The average number of premature responses of the overall group stabilized at ~30% in the last 4 sessions of training.
- *Test* The VDS testing session occurred on a single day and consisted of 120 trials, similar to those previously described, with the exception of the delay, which was 3 s in the first and the last 25 trials and randomly either 6 or 12 s in the middle 70 trials (leading to a 3 s – 6/12 s – 3 s configuration), and, importantly, the fact that multiple nose pokes were allowed during the delay period, i.e., premature responses did not initiate TO punishment periods. (Figure 1B).

### 5-choice serial reaction time task

The 5-csrtt was performed in the same apparatus as the VDS, following the general principles originally described by (Carli et al., 1983). Briefly, each session started with the delivery of one pellet. Its collection by the animal initiated the first trial. At this point the house light is on signaling an ongoing trial. After an ITI of 5 s one of the five lights in the rear panel was illuminated for a period of 60 S. Nose pokes in this aperture during the light period or in the subsequent 5 s were rewarded with one pellet in the food aperture whose collection marks the beginning of an ITI that precedes a new light signal. Nose pokes in any of the other 4 apertures initiated a time-out period of 5 s in darkness after which a new trial is started (house light on). Each session consisted in a sequence of 100 trials (or a maximum of 30 min) performed twice a day during the morning/afternoon periods. The performance of the subjects was assessed using the following experimental parameters:

- Accuracy—ratio of correct/total number of responses.
- Prematurity—responses during the ITI in any of the five apertures (triggers a TO).
- Omissions—absence of response in appropriate time.

Other parameters including latency-to-feed and response delay were also registered. Throughout the sessions the level of difficulty was increased by decreasing the stimulus duration from 60 to 30, 10, 2, and 0.5 S. An accuracy ≥80% and omissions ≤20% in two successive sessions were considered as criteria for level change. The last level was performed during 15 sessions. The 5-csrtt was initiated 2 days after the last session of the VDS; no training preceded the sessions.

### Delay-discounting

The DD task was performed in (OCs; 30.5 cm L × 24.1 cm W × 21.0 H) equipped with two retractable levers located on either side of a food magazine and a house light placed in the opposite side (MED Associates). Information regarding animals' activity within the OC was registered with MED-PC IV software. Two chambers were used each placed within an individual sound attenuating cubicle. In the first 2 days, animals were placed in the OCs for 5 min with the house light on and both levers retracted. In the food magazine 3–5 pellets were made available. From days 3–5 a continuous reinforcement protocol (CRF) was applied. A single lever was made available and a sugared pellet was delivered for each lever press. Sessions were terminated when 50 pellets were obtained or if 30 min had elapsed and were immediately followed by a similar session differing only in the fact that the levers were switched. The lever presentation order was counterbalanced over days 3–5. The second step of the training protocol consisted in 3 sessions (1 per day; days 6–8) on which the animal was required to nose poke the food magazine in order to trigger the lever presentation and initiate the trial when the house light was on. Only one lever was presented at each trial in a random and balanced fashion (i.e., left and right levers were presented an equal number of times) up to 90 trials with a fixed duration of 70 S. The DD proper sessions consisted in 4 blocks of 10 trials each with an organization similar to that described for training days 6–8 differing in that the nose poke in this case triggers the simultaneous presentation of both levers. One lever is now associated with a small (1 pellet) but immediate reward and the other with a large (4 pellets) but delayed reward (delays: 0, 15, 30, and 45 s respectively in the 1st, 2nd, 3rd, and 4th blocks). The value attributed to each of the two levers is balanced between animals. Each block of 10 trials is preceded by two forced-choice trials in which each lever is individually presented and the pellets are delivered according to the respective block parameters. The DD sessions were repeated uninterruptedly for 20 days and the animals that maintained a robust selection of the favorable lever in the 1st block—5 consecutive days with preferences over 70%—were selected for analysis (*N* = 8). The area under the curve (AUC) was used as the main measure of impulsive DD, but data were also analyzed according to the exponential or the hyperbolic functions (Odum, 2011).

### Statistical analysis

Data is presented as mean ± SEM and analyzed using 1- or 2-way analysis of variance followed by a *post-hoc* (Tukey) for multiple comparisons. Independent-samples *t*-test was used to test for the drug effect within each delay. Intra-individual comparisons in different paradigms were performed by linear regression analyses. Results were considered statistically significant if *p* < 0.05.

## Results

### Pharmacological validation

**Figure 2:**
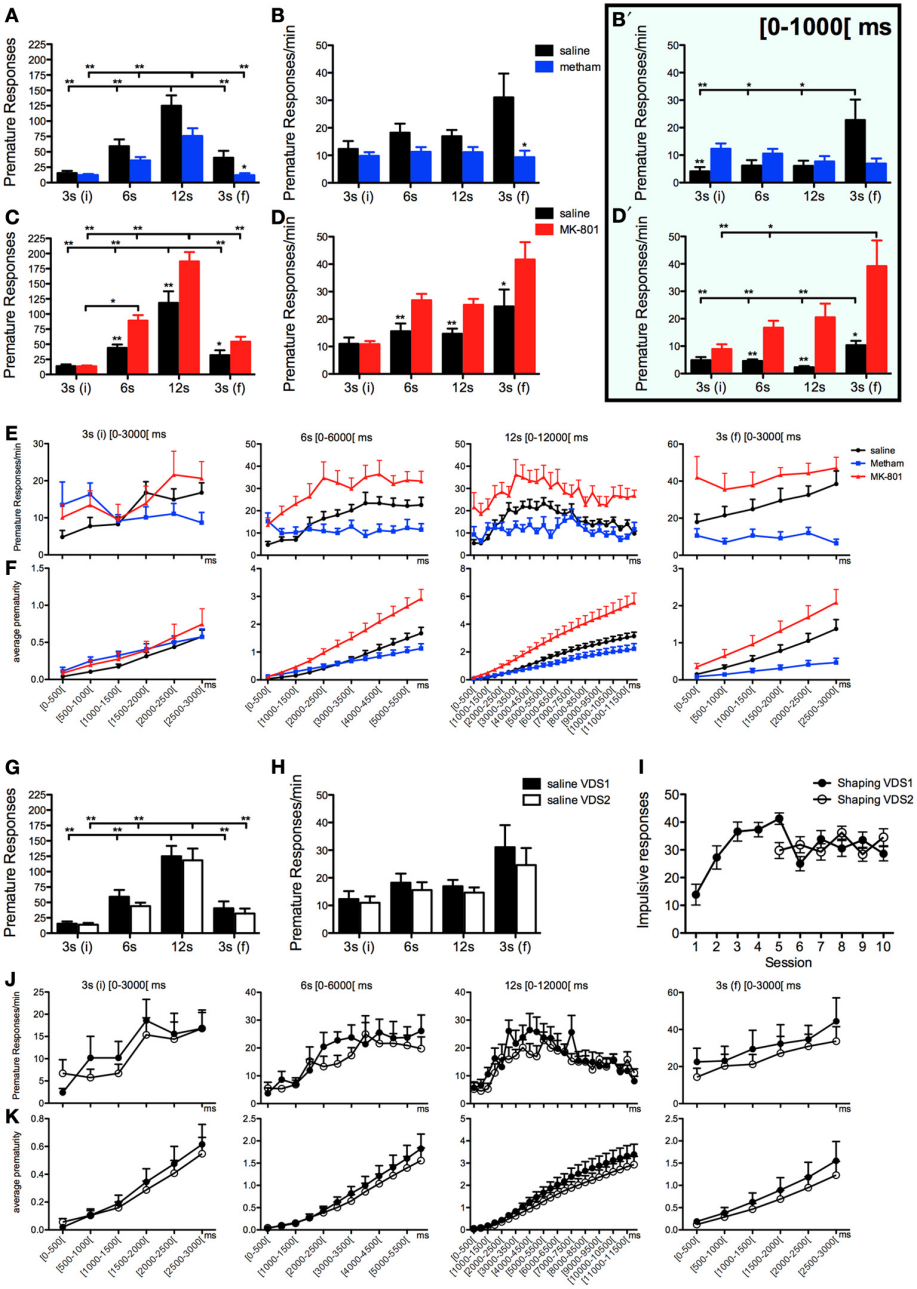
**Pharmacological validation of the VDS with methamphetamine and MK-801. (A–F)** Methamphetamine (VDS1) and MK-801 (VDS2) decreased and increased, respectively, the absolute number of premature responses **(A, C)** and the number of premature responses per available minute of delay **(B, D)**. In the case of methamphetamine, but not MK-801, this pattern was inverted in the initial second of the 3 s (i) delay segment **(B′, D′)**. The absolute **(E)** and the accumulated **(F)** average prematurity/trial response profile is presented in a segmented (500 ms periods) fashion for each delay segment 3 s (i), 6, 12, and 3 s (f). The same analyses are presented to compare the saline controls in VDS1 and 2 experiments **(G–K)**. No differences were observed between VDS 1 and 2 nor between the impulsive behavior in the preceding training sessions **(I)** indicating that the VDS permits multiple tests without significant alterations of the basal behavior. Statistically significant comparisons between delay segments are marked with a horizontal line over the relevant graph bars; statistically significant comparisons between groups are marked over the graph bar of lowest value. ^*^P < 0.05; ^**^P < 0.01; data presented as mean + S.E.M. 5-csrtt—5 choice serial reaction time task; DD—delay discounting; VDS—variable delay-to-signal task.

We tested the ability of the VDS to discriminate impulsive behavior in conditions known to decrease or increase impulsivity. As expected, acute challenge with methamphetamine (VDS1) diminished, whereas acute treatment with MK-801 (VDS2) augmented, the absolute number of impulsive responses (IR) [VDS1: *F*_(1, 16)_ = 5.416 *p* = 0.033; VDS2: *F*_(1, 18)_ = 23.258 *p* < 0.001] (Figures 2A–D). Additionally, although the number of premature responses varied in accordance with the delay in both experiments [VDS1: *F*_(3, 48)_ = 90.248 *p* < 0.001; VDS2: *F*_(3, 54)_ = 254.888 *p* < 0.001], this effect was stronger in the saline group as compared to the methamphetamine group [VDS1: *F*_(3, 48)_ = 5.478 *p* = 0.016] and in the MK-801 compared with the respective saline group [VDS2: *F*_(3, 54)_ = 26.066 *p* < 0.001] (Figures 2A,C). Importantly, the ability of the protocol to detect changes in impulsivity was further confirmed by an analysis of response rates (responses per minute of delay–IR/m), which were decreased by methamphetamine treatment and increased by acute MK-801 injections [VDS1: *F*_(1, 16)_ = 4.815 *p* = 0.043; VDS2: *F*_(1, 18)_ = 17.449 *p* = 0.001]. Interestingly, this analysis also revealed that although animals from all groups kept their premature response rate approximately constant across the 3 delay blocks, they increased it in the last 3 s delay [VDS1: *F*_(3, 48)_ = 6.931 *p* = 0.011; VDS2: *F*_(3, 54)_ = 28.166 *p* < 0.001], an effect that was present in the saline group but not in the methamphetamine group [VDS1: *F*_(3, 48)_ = 8.767 *p* = 0.005] and was stronger in the MK-801 compared with the respective saline group [VDS2: *F*_(3, 54)_ = 12.973 *p* < 0.001] (Figures 2B,D).

To further explore the possibilities offered by VDS in the characterization of impulsivity, we decided to partition data from each delay block into 500 ms intervals, the results of which (pooling data from VDS1 and VDS2 together) are depicted in Figures 2E,F. Overall, this analysis was in line with our previous data in that methamphetamine treated animals showed decreased, whereas animals treated with MK-801 had increased, premature responses and response rates compared with saline treated animals, particularly near the end of each delay block and more obvious across the entire final delay block [3 s(f)]. This way of looking at our data also revealed an alteration to the overall response pattern, specifically of methamphetamine treated animals, in the initial 1000 ms of delay, which prompted us to analyze premature response rates on this initial period across delays and treatment groups (Figures 2B′,D′). In this initial period of each delay block, MK-801 maintained its pro-impulsivity effect [*F*_(1, 18)_ = 13.780 *P* = 0.002] whereas methamphetamine failed to reduce impulsivity [*F*_(1, 16)_ = 0 *P* = *ns*], with pairwise comparisons even revealing a significant increase in response rates of methamphetamine treated animals in the first (3 s) delay block (*t*_16_ = −3.180 *p* = 0.006). However, in line with results from the entire delay period, data from the first 1000 ms also showed that methamphetamine treatment, but not MK-801, prevents the increase in premature response rates observed in the last delay block [3 s(f)] of the other experimental groups [VDS1: delay *F*_(3, 16)_ = 5.163 *P* = 0.012 drug′delay *F*_(3, 48)_ = 7.533 *P* = 0.002; VDS2: delay *F*_(3, 18)_ = 11.918 *P* < 0.001 drug^*^delay *F*_(3, 54)_ = 3.209 *P* = 0.063] (Figures 2B′,D′).

**Figure 3:**
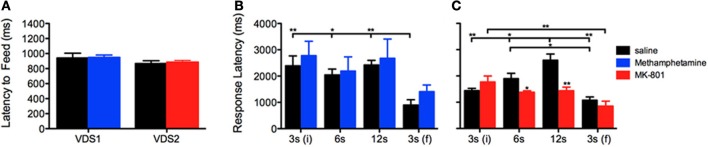
**Methamphetamine and MK-801 influence on (A) latency to feed and (B,C) response latency measures.** Statistically significant comparisons between delay segments are marked with a horizontal line over the relevant graph bars; statistically significant comparisons between groups are marked over the graph bar of lowest value. ^*^P < 0.05; ^**^P < 0.01; data presented as mean + S.E.M. VDS—variable delay-to-signal task.

To test the presence of a putative effect of VDS repetition on impulsive behavior (Figures 2G–K), we compared the performance of the saline groups in VDS1 and VDS2. Apart from confirming a significant effect of the delay block both in the number of responses, that varied according to the amount of delay, [*F*_(3, 48)_ = 193.262 *P* < 0.001] and in the response rate, which was constant in the first 3 delay blocks but increased dramatically in the last (3 s) period [*F*_(3, 48)_ = 12.460 *P* = 0.001], we could not find any significant effect of experiment on either parameter [number of responses VDS1 vs. VDS2: *F*_(1, 16)_ = 2.607 *P* = *ns*; response rate VDS1 vs. VDS2: *F*_(1, 16)_ = 2.468 *P* = *ns*] nor any interaction between experiment and the effects of delay block described above [number of responses experiment*delay: *F*_(3, 48)_ = 2.660 *P* = *ns*; response rate experiment^*^delay: *F*_(3, 48)_ = 3.422 *P* = *ns*] (Figures 2G,H). Similarly, the response pattern along the delay blocks was similar in both saline groups (Figures 2J,K). Importantly, methamphetamine and MK-801 at the selected doses had no sedative or motivational effects, as no differences were observed in the latency to feed (Figure 3A). On the contrary, the two drugs had contrasting effects in response latency. While methamphetamine treatment had no effect in this parameter [*F*_(1, 16)_ = 0.002 *P* = *ns*] and failed to abrogate a decrease in response latency in the last delay block [3 s(f)] [delay: *F*_(3, 48)_ = 14.508 *P* < 0.001; drug^*^delay: *F*_(3, 48)_ = 2.494 *P* = *ns*], MK-801 had a profound effect, not only shortening response latencies [*F*_(1, 18)_ = 7.011 *P* = 0.016] but also reducing the influence of delay block on them [delay: *F*_(3, 54)_ = 24.352 *P* < 0.001; drug^*^delay: *F*_(3, 54)_ = 10.403 *P* < 0.001]. (Figures 3B,C).

### Comparisons with reference paradigms

**Figure 4:**
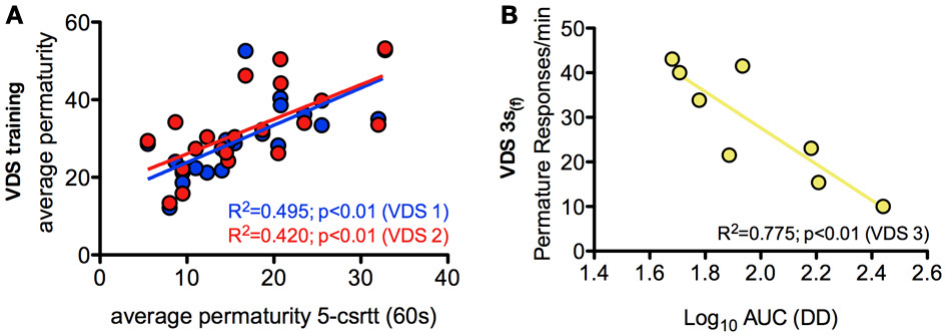
**Relevant correlations between 5-csrtt and DD performances and the VDS. (A)** The average number of premature responses in the 60 s stage of the 5-csrtt correlated with the number of premature responses of both VDS 1 and VDS 2 training periods. **(B)** Preference for delayed choices (as measured by the AUC) in DD was inversely correlated with premature responses in the 3 s (f) period of the VDS. See Table 1 for complete analyses. 5-csrtt—5 choice serial reaction time task; AUC—area under the curve; DD—delay discounting; VDS—variable delay-to-signal task.

In order to further characterize the profile of impulsivity assessed by the VDS, we compared it against reference paradigms, namely the 5-csrtt (in experiment 1) and DD (in experiment 2). The parameters used to correlate assessments of impulsivity of the same animal made in different paradigms were: i) the average number of impulsive responses in the last 5 days of training before VDS, a period where impulsive responses were stable (Figure 2I), ii) the average rate of impulsive responses per minute of delay, partitioned in segments of 3 S, during the VDS, iii) the average number of impulsive responses in the different stages of the 5-csrtt - 60, 30, 10, 2, and 0.5 s of signal duration—and iv) the AUC in the DD. While comparisons 5-csrtt and training included all animals of experiment 1, only animals injected with saline were used for comparisons with VDS 1 and 2 (Figure 4). The number of impulsive responses in the training protocol of both VDS 1 and 2 was strongly correlated with the first stage of the 5-csrtt, but not with later stages of increasing attentional demand (Figure 4; Table 1). On the contrary, the best correlate of the rate of impulsive responses in the VDS was performance in the 10 s stage of the 5-csrtt, but not in periods with shorter or longer stimulus presentations (Figure 4; Table 1). Finally, the rate of premature responses per min in the last delay block of the VDS (as well as in part of the 12 s delay) was negatively correlated with the AUC of the DD (of note, in this test a smaller area corresponds to a higher impulsivity) (Figure 4; Table 1). Importantly, this significant correlation also holds true when using the coefficients derived by fitting an exponential or hyperbolic function to the response curve.

**Table 1:** **Linear regression of the comparisons between VDS and standard impulsivity paradigms: 5-csrtt and DD**.

**5-csrtt**		**Training**		**3 s(i) [0–3] s**		**6 s [0–3] s**		**6 s [3–6] s**		**12 s [0–3] s**		**12 s [3–6] s**		**12 s [6–9] s**		**12 s [9–12] s**		**3 s(f) [0–3] s**	
		**VDS 1**	**VDS 2**	**VDS 1**	**VDS 2**	**VDS 1**	**VDS 2**	**VDS 1**	**VDS 2**	**VDS 1**	**VDS 2**	**VDS 1**	**VDS 2**	**VDS 1**	**VDS 2**	**VDS 1**	**VDS 2**	**VDS 1**	**VDS 2**
**60 s**	**R2**	0.495	0.420	0.164	0.274	0.159	0.043	0.057	0.002	0.234	0.097	0.055	0.061	0.110	0.004	0.052	0.007	0.057	0.018
	***P***	**0.001**	**0.002**	0.319	0.121	0.327	0.563	0.570	0.897	0.225	0.382	0.576	0.490	0.423	0.871	0.586	0.819	0.567	0.715
**30 s**	**R2**	0.044	0.042	0.043	0.239	0.216	0.036	0.164	0.005	0.213	0.087	0.073	0.276	0.121	0.198	0.119	0.159	0.162	0.011
	***P***	0.375	0.388	0.622	0.152	0.246	0.601	0.320	0.849	0.250	0.409	0.518	0.119	0.398	0.198	0.402	0.253	0.323	0.774
**10 s**	**R2**	0.088	0.102	0.185	**0.701**	0.290	**0.614**	**0.703**	0.179	**0.700**	**0.733**	0.417	0.214	**0.549**	**0.561**	0.461	0.277	0.486	0.194
	***P***	0.203	0.170	0.288	**0.003**	0.168	**0.007**	**0.009**	0.223	**0.010**	**0.002**	0.084	0.178	**0.035**	**0.013**	0.064	0.118	0.055	0.203
**2 s**	**R2**	0.003	0.014	0.057	0.052	0.414	0.275	0.311	0.161	0.376	0.241	0.363	0.159	0.402	0.248	0.325	0.220	0.402	0.106
	***P***	0.833	0.621	0.569	0.526	0.085	0.120	0.151	0.251	0.106	0.150	0.114	0.254	0.091	0.143	0.140	0.172	0.092	0.358
**0,5 s**	**R2**	0.000	0.001	0.001	0.330	0.086	0.149	0.183	0.096	0.197	0.208	0.100	0.179	0.141	0.239	0.123	0.289	0.189	0.111
	***P***	0.994	0.925	0.931	0.082	0.480	0.271	0.291	0.383	0.270	0.186	0.445	0.224	0.360	0.152	0.394	0.109	0.282	0.347
**DD**				**3 s(i) [0–3] s**		**6 s [0–3] s**		**6 s [3–6] s**		**12 s [0–3] s**		**12 s [3–6] s**		**12 s [6–9] s**		**12 s [9–12] s**		**3 s(f) [0–3] s**	
				**VDS3**		**VDS3**		**VDS3**		**VDS3**		**VDS3**		**VDS3**		**VDS3**		**VDS3**	
**AUC**	**R2**			0.432		0.237		0.389		0.198		0.409		**0.763**		0.172		**0.755**	
	***P***			0.077		0.221		0.098		0.270		0.088		**0.010**		0.307		**0.005**	
**K^1^**	**R2**			0.436		0.097		0.350		0.029		0.418		0.609		0.407		**0.810**	
	***P***			0.328		0.836		0.441		0.95		0.351		0.146		0.365		**0.027**	
**K^2^**	**R2**			0.532		-0.053		0.339		−0.147		0.547		0.643		0.501		**0.807**	
	***P***			0.219		0.91		0.458		0.753		0.204		0.119		0.252		**0.028**	

Concerning the later, comparisons were made with the AUC and with the hyperbolic/exponential decaying coefficients, k^1^ and k^2^, respectively. Significant comparisons (P < 0.05) are highlighted in bold.

## Discussion

The VDS paradigm was designed according to some simple principles: (i) The task (and its training protocol) is performed in a standard 5-csrtt apparatus in which only the center nose poke aperture is available. Such approach has been tried before by Dalley and colleagues (2002) and is intended to increase the efficiency of the task by reducing the attentional load. (ii) The training protocol consists in twice daily sessions on a “differential-reinforcement-of-low-rate” (DRL)-like schedule that quickly (4 days) achieve stability and a high degree of learning. Of notice, premature responses under DRL are often considered a measure of impulsivity, particularly under stable schemes such as ours (Pizzo et al., 2009) and can even be conceptualized as a delay discounting (Monterosso and Ainslie, 1999) which, although not the focus of the present paper, can also contribute to enrich the assessment of impulsivity obtained with the VDS. (iii) The initial block of 25 trials with 3 s delays until light presentation establishes a baseline against which results of the other blocks can be compared and assesses the acquisition of the training protocol. In our pharmacological assays, baseline behavior did not differ between saline and methamphetamine (VDS1) or MK-801 (VSD2) groups. (iv) The following block of 70 trials exposes animals to larger delays of 6 and 12 s, randomly presented. Importantly, this probably induces two sources of behavioral control, similarly to what happens in mixed-fixed interval (FI) experiments (Whitaker et al., 2003), that might contribute to the increased responding observed in the final 3 s-delay block (Baron and Leinenweber, 1995). (v) The last block consists of 25 trials with a delay of 3 s before light presentation, in which control animals present an increased response rate; this is in accordance with current concepts in behavioral timing, in which the rate of responding has an inverse relationship with the duration of the interval (Kirkpatrick, 2002; Guilhardi et al., 2005).

An approach similar to ours, a go/no-go task using delays of variable duration (9 to 24 s) has been already described by Mitchell and co-workers (Gubner et al., 2010; Moschak and Mitchell, 2012). However, while the variable delays constitute the core of their task, from which measures of impulsive behavior are taken, the variable intervals in the VDS act as a trigger of increased impulsivity between the basal 3 s block and the final 3 s block, contributing to unmask manifestations of impulsive behavior. In addition, while the VDS can be conducted in a single session after a training protocol of fixed duration (10 sessions, 5 days), the total number of training sessions in the previously described task can amount to 11 days, depending on each animal's performance.

In a first attempt to characterize VDS, we assessed animals acutely treated with MK-801 or methamphetamine, two drugs with well described and opposite effects on impulsive behavior. Supporting its validity as a test of impulsivity, acute MK-801 treatment (VDS2), which induces enhanced impulsivity (Fletcher et al., 2011), caused a generalized increase in the number and rate of premature responses across all delay blocks, whereas acute challenge with methamphetamine (VDS1), which acts as a stabilizer of impulsive decisions in animals (Richards et al., 1999; Winstanley et al., 2003) and humans (De Wit et al., 2002), prevented the increase in premature response rates displayed by control animals in the last (3 s) block. Interestingly, the latter results seem to be critically dependent on the existence of a previous block of randomly presented 6/12 s delays in our protocol, since it was shown that only variable delays, as opposed to fixed delays, trigger the inhibitory action of acute methamphetamine upon premature responses on a FI protocol (Hayton et al., 2012). Besides this effect on the last block, metamphetamine treatment also resulted in a completely stable response profile across blocks, independently of the delay. Of notice, this enhanced delay tolerance is in accordance with data from DD tasks in which acute amphetamine induces a delay insensitive behavior (Winstanley et al., 2003).

To further characterize VDS, we correlated performance in our test with results of the same animals in one of two standard paradigms of impulsivity (Winstanley et al., 2010; Dalley et al., 2011; Dalley and Roiser, 2012): the 5-csrtt (VDS1) and the DD (VDS2). For these comparisons, VDS delays were divided in bins of 3 s for several reasons: (i) all delays were multiple of 3 and therefore this was a convenient option for analyses; (ii) the training protocol was set at a periodicity of 3 S/trial and therefore this could be considered the basal value of delay tolerance; (iii) we observed that the impulsivity response profile along the delay was not stable and varied in periods of ~3 s (see Figure 2E).

The strong positive correlation between impulsive behavior in the initial stage of the 5-csrtt and the training period preceding the VDS fits with the fact that both tests use a DRL-like schedule, in which premature responses lead to a time-out period. In contrast, there were almost no significant correlations between impulsivity behavior in the 5-csrtt and the VDS, which likely relates to the fact that, in contrast to the training period, premature responses in the VDS are not “punished.” Indeed, this protocol difference implies that parameters used as measures of impulsivity in both tests are of a fundamentally different nature (percentage of prematurely interrupted trials in the 5-csrtt vs. number/rate of premature responses in the VDS) and probably correspond to different types of impulsivity. This idea is supported by the fact that methamphetamine administration decreased impulsivity in the VDS (present study) and decision impulsivity paradigms, including the DD (Richards et al., 1999; Winstanley et al., 2003), but increased response impulsivity in the 5-csrtt (Cole and Robbins, 1987; Fletcher et al., 2011). In line with this, and despite its overall inhibitory action, is the observation that metamphetamine increased the rate of premature responses in the first second of the delay period might reflect increased response impulsivity. More importantly, the rate of premature responses in the final 3 s block of the VDS was strongly and significantly correlated with preference for delayed choices in the DD, either quantified by the AUC or by an equivalent parameter in terms of hyperbolic or exponential discounting functions (Odum, 2011). As the latter is the gold-standard in decision impulsivity assessment, this, together with data on metamphetamine and MK-801 discussed above, strongly supports the validity of VDS as a test of impulsive behavior.

The VDS presents a number of advantageous characteristics over the available impulsivity paradigms: (i) It has a significantly shorter training period (10 twice daily sessions) when compared with the ≈35–55 days of 5-csrtt and DD (Winstanley, 2011), (ii) it requires only one test session, (iii) it is resistant to multiple testing (has almost no test-retest effect) making it particularly suitable for longitudinal assays. Our validation assays, namely in the comparisons with the 5-csrtt and DD, relied in the intrinsic behavioral variability of an outbred population (Wistar Han), and not in artificially induced variability (e.g., by drug treatments or genetic manipulations), reinforcing the sensitivity of our paradigm in terms of impulsivity assessment. It should, however, be stated that the VDS does not replace paradigms like the 5-csrtt, where impulsivity is measured in conditions of high attention demand or like the DD, where an actual choice is presented. Recently, we have used an earlier version of the VDS to successfully demonstrate alterations in impulsive behavior in animals with neuropathic pain (Leite-Almeida et al., 2012).

### Conflict of interest statement

The authors declare that the research was conducted in the absence of any commercial or financial relationships that could be construed as a potential conflict of interest.
